# Magnitude of the Regression to the Mean within One-year Intra-individual Changes in Serum Lipid Levels among Japanese Male Workers

**DOI:** 10.2188/jea.11.61

**Published:** 2007-11-30

**Authors:** Yutaka Takashima, Yuu Sumiya, Akatsuki Kokaze, Masao Yoshida, Mamoru Ishikawa, Yasuko Sekine, Syoko Akamatsu

**Affiliations:** 1 Department of Public Health, Kyorin University School of Medicine.; 2 Saison Health Care Center.

**Keywords:** serum lipids, regression to the mean, health check-up, longitudinal data

## Abstract

To investigate the magnitude of the ‘regression to the mean’ effect for the changes in serum total cholesterol (TC), triglycerides (TG), and high density lipoprotein cholesterol (HDLC) levels during one-year interval between annual health check-ups in occupational settings, the relationships between the baseline level and subsequent one-year change in TC, TG or HDLC were analyzed using paired health check-up data in 1998 and 1999 of 547 Japanese male clerical workers.

After adjustment for age, body mass index (BMI), yearly change in BMI, drinking score and smoking score by the multivariate analyses, the yearly changes in each serum lipid (ΔTC, ΔLn(TG) or ΔHDLC) were clearly inversely associated with the lipid levels in 1998. For example, in the multiple linear regression analyses setting Δ value in each serum lipid as a dependent variable, the partial regression coefficients for the baseline lipid levels (β_1_) were - 0.21 (p<0.001) for the TC, - 0.39 (p<0.001) for the Ln(TG) and - 0.15 (p<0.001) for the HDLC, respectively.

These results suggest that the observed yearly change in each serum lipid level may largely reflect the ‘regression to the mean’ effect in addition to the real yearly biological change.

## INTRODUCTION

It is well known that the range of measured values of risk factors is generally exaggerated during an initial baseline survey and decreases as the observation period proceeds in a prospective study^1^^, ^^2^^)^. The combined effects of measurement errors and biological fluctuations or changes within persons cause this phenomenon, leading to the production of ‘regression dilution bias’^3^^-^^5^^)^. When the measured value of a bio-marker shows a symmetric distribution with one peak such as the Gaussian distribution, these effects raise the measured value above the ‘true’ value if the measured value exceeds the mean value of the population, while reducing the measured value below the ‘true’ one if the measured value is located under the mean. Therefore, the measured levels at the baseline show the regression to the mean of the population as the observation period proceeds. In order to appropriately evaluate the measured risk factor levels at the baseline or their changes after a particular interval, it is potentially important to make relevant adjustment for such ‘regression to the mean’ effect. Some previous reports in Western countries well illustrate the influences of this effect using the examples of blood pressure^1^^, ^^5^^)^ or serum total cholesterol (TC) level^1^^, ^^3^^, ^^4^^)^. However, the magnitude of this effect has not yet been elucidated in serum triglyceride (TG) or HDL cholesterol (HDLC) levels. And in Japan, there have not been epidemiological reports on this effect. Therefore, in the present study, the magnitude of the ‘regression to the mean’ effect for one-year change in each of TC, TG and HDLC levels was investigated using annual health check-up records for Japanese male workers. The results of this study will contribute to the appraisal of the regression dilution bias for risk assessment in a cohort study setting serum lipid levels as risk factors at baseline in Japan.

## METHODS

### Subjects

The subjects of this study were Japanese male clerical workers of a sales company located in the metropolitan area of Japan. At the company the periodic health check-ups had been conducted on May every year, and the medical and life-style records of 727 subjects obtained from the health check-ups in 1998 and/or in 1999 were used as the materials of the present study. The content of the health check-up records comprised height and weight, blood pressure, serum total cholesterol (TC), triglycerides (TG) and high density lipoprotein cholesterol (HDLC) levels, hematological findings, urine findings, past history, present status of disease control or treatment, subjective and objective symptoms, drinking history, smoking history, etc. Among these records, those of 547 subjects who had participated in the health check-ups in both years and had not received any medications for hyperlipidemia in these two years were actually used for the present analysis.

### Items for the study

In both years, all the blood samples were collected by venous puncture under the condition of overnight fasting (that is, collection after at least 12 hours fasting). The laboratory where the quantification of serum TC, TG and HDLC was conducted, had been periodically examined for the accuracy of the measurements by the Japan Medical Association. And both of the results from the two quality control surveys in 1998 and 1999 showed the excellence (class ‘5’ (= ‘excellent’) by the fifth grade evaluation for each of serum TC, TG and HDLC levels) of validity and reproducibility of the measurement value in serum TC, TG or HDLC from the tests using the control serum. However, because the measurement of a control serum was not included in both health check-ups, the magnitude of the systematic error of measurement between 1998 and 1999 could not be elucidated. Serum TC concentration was determined by the enzymatic method^6^^)^. The determinations of TG and HDLC levels in serum were performed by the colorimetric methods using glycerokinase, and peroxydase for the TG^7^^)^ and a nonionic surfactant, cholesterolesterase and cholesteroloxydase for the HDLC^8^^)^.

For the present study, body mass index (BMI) was calculated as an indicator of obesity index using the following formula: BMI= body weight (Kg) /( height (m))^2^

As tor drinking history, according to drinking frequency records from the health check-up data in 1998, the 547 subjects were categorized into the following four groups: ‘non-drinkers’, ‘one to three times per week’, ‘four to six times per week’, and ‘everyday’. Then, for the analysis these four categories were scored as ‘0’, ‘1’, ‘2’, and ‘3’, respectively. Smoking history was classified into the following three groups according to Brinkman Index (BI)^9^^)^ in 1998: ‘non-smoker (BI=0)’, ‘0<BI<400’, and ‘BI≥400’. Then, just like the scoring of drinking history, these three groups were scored as ‘0’, ‘1’, and ‘2’, respectively. Of the 547 subjects, the numbers of subjects corresponding to the score ‘0’, ‘1’, ‘2’, and ‘3’ in the drinking history, were 89, 165, 114 and 179, respectively. The numbers of the smoking score ‘0’, ‘1’, and ‘2’, were 251, 129 and 167, respectively.

### Statistical analyses

The distributions of TC and HDLC levels in 1998 for the 547 subjects were similar to the Gaussian distribution, although the distribution modes were not exactly symmetric. However, the distribution of TG level was markedly skewed from the Gaussian distribution toward a lognormal distribution. Therefore, in the statistical procedures of this study, TG level was converted to the natural logarithm value (Ln(TG)).

Since any ‘true’ values of these lipid levels were unknown in this study, only method to ascertain the ‘regression to the mean’ effect was to confirm the shrinkage of the range in these lipid levels during one-year period among the subjects. The shrinkage of the range can be verified by the ascertainment of inverse association between one-year change in a serum lipid level and the baseline level of the lipid. Thus, in order to explore the ‘regression to the mean’ effect, firstly, the mean values of TC, Ln(TG) and HDLC in 1998 were compared with those in 1999 by the quintiles of baseline levels of each serum lipid (TC, Ln(TG) and HDLC levels in 1998). By this procedure, it was confirmed whether the shrinkage of the range between the mean values in the top and bottom quintiles could be observed. Secondly, after the yearly changes in the TC, Ln(TG) and HDLC (ΔTC, ΔLn(TG) and ΔHDLC) levels were calculated for each subject, the associations between the yearly change and the baseline level were explored for each serum lipid. For this objective, in addition to the calculation of simple regression coefficients by the simple regression analysis, two types of multiple linear regression models were utilized. Both models set the yearly change in each serum lipid as a dependent variable and included age in 1998, BMI in 1998, yearly BMI change between 1998 and 1999, drinking score and smoking score as independent variables. However, while in one model the categorization number for quintiles of each serum lipid in 1998 (1, 2, 3, 4, and 5) was included as an independent variable in addition to the above four variables, in the other model the serum lipid level in 1998 instead of the categorization number for quintiles was included into the independent variables. That is, the equations of these two models were as follows:

Δ value of a serum lipid= *α* + β_1_ × (the categorization number for quintiles of the lipid level in 1998) + β_2_ × (age in 1998) + β_3_ × (BMI in 1998) + β_4_ × (ΔBMI) + β_5_ × (drinking score) + β_6_ × (smoking score)

and

Δ value of a serum lipid= *α* + β_1_ × (the lipid level in 1998) + β_2_ × (age in 1998) + β_3_ × (BMI in 1998) + β_4_ × (ΔBMI) + β_5_ × (drinking score) + β_6_ × (smoking score)

where *α*: intercept, β_1-6_ : partial regression coefficients

From the former model, the adjusted means (least square means)^10^^)^ of the yearly change in each serum lipid could be calculated by the quintiles of each serum lipid in 1998. From the latter model, the magnitude of the change in Δ value due to one unit (1mg/dl) change in the baseline level of the serum lipid could be indicated by the partial regression coefficient of serum lipid level in 1998 (β_1_). All these statistical analyses were performed using SAS statistical package^11^^)^. The PROC GLM^12^^)^ was applied for the analyses using the aforementioned two models.

## RESULTS

The health check-up data in 1998 and those in 1999 among the 547 subjects are summarized and compared in Table 1. It was found that the mean TC level decreased by 4.7mg/dl during one year, whereas the arithmetic and geometric mean TG levels (similarly the mean Ln(TG) level) slightly increased. While the BMI level, and systolic and diastolic blood pressure increased, the HDLC level decreased.

**Table 1.** Comparison of paired health check-up data (data in 1998 and those in 1999) among 547 subjects.

	Data in 1998	Data in 1999
Age^†^	43.2 (7.7)	44.2 (7.7)
Age-range	25-59	26-60
BMI (Kg/m^2^)^†^	23.2 (2.9)	23.3 (2.9)
TC (mg/dl)^†^	205.4 (33)	200.7 (33.5)
TG (mg/dl)^†^	155.2 (127)	158.7 (109.2)
TG (mg/dl)^¶^	126.4 (1.8)	133.0 (1.8)
Ln (TG) (mg/dl)^†^	4.84 (0.6)	4.89 (0.58)
HDLC (mg/dl)^†^	54.4 (13.6)	52.3 (13.7)
SBP (mmHg)^†^	127.8 (17.1)	129.9 (17)
DBP (mmHg)^†^	79.3 (11.9)	82.8 (13.1)

Mean and SD (in parentheses) are listed except for “Age-range”.

†: Arithmetic mean and SD, ¶: Geometric mean and geometric SD

BMI : Body mass index, TC : Total cholesterol level,

TG : Triglycerides level, Ln(TG) : Natural logarithm of triglycerides level,

HDLC : High-density-lipoprotein cholesterol level

SBP : Systolic blood pressure, DBP : Diastolic blood presuure

The correlation coefficients between 547 pairs of measurements (the measured value in 1998 and that in 1999) were 0.77 for the TC, 0.68 for the Ln(TG), and 0.86 for the HDLC, respectively.

**Figure 1.**
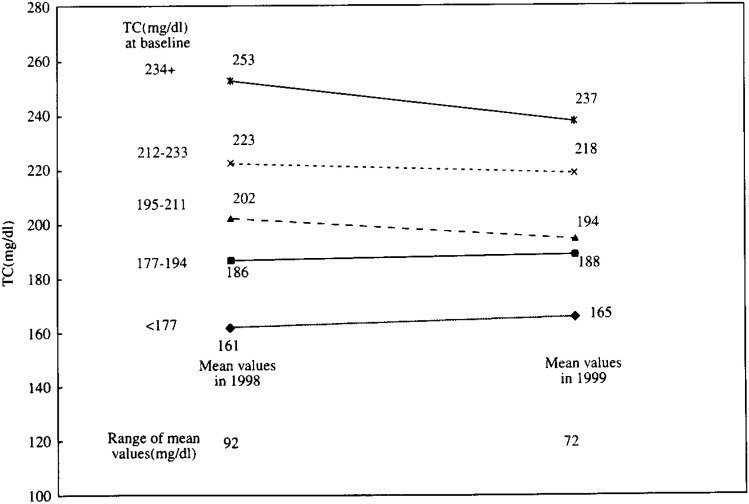
Comparison of mean TC in 1998 and those in 1999 by quintiles of TC in 1998. TC : Total cholesterol level.

**Figure 2.**
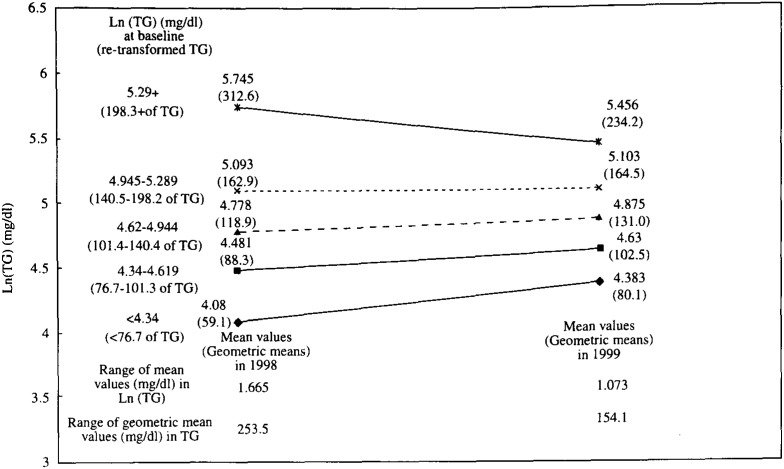
Comparison of mean Ln(TG) and geometric mean of TG (in parentheses) in 1998 and those in 1999 by quintiles of Ln(TG) in 1998. TG : Triglycerides level, Ln(TG) : Natural logarithm of triglycerides level.

**Figure 3.**
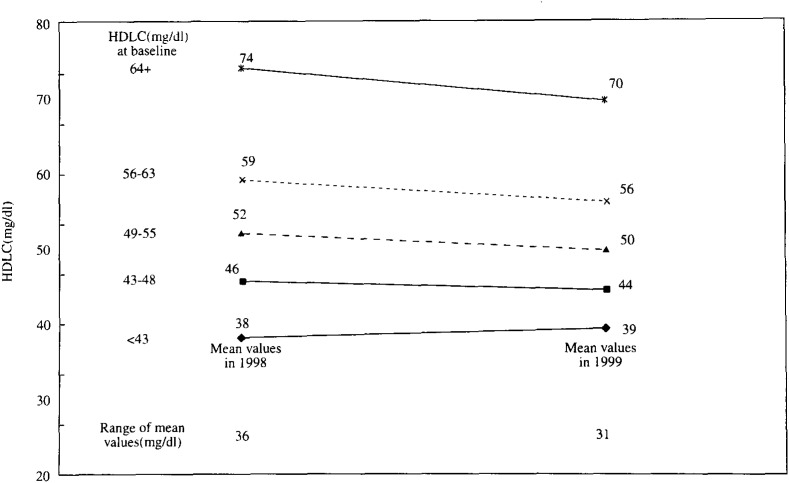
Comparison of mean HDLC in 1998 and those in 1999 by quintiles of HDLC in 1998. HDLC : High - density - lipoprotein cholesterol level.

In Fig. 1, 2 and 3, the mean values of TC, Ln(TG) and HDLC in 1999 are shown in contrast to those in 1998 by the quintiles of each serum lipid level in 1998. These figures demonstrate that the range between the mean values in the top and bottom quintiles clearly shrank after one-year interval in these three lipids. For example, the mean TC levels of persons who were in the top quintile initially (i.e. those with a TC value of 234 mg/dl or more at the baseline) declined from 253 mg/dl in 1998 to 237 mg/dl in 1999. Similarly, the mean TC levels of persons in the bottom quintile initially (i.e. those with a value of less than 177 mg/dl at the baseline) increased from 161mg/dl in 1998 to 165 mg/dl after one year. As a result, the range between the mean values in the top and bottom quintiles declined from 92 mg/dl in 1998 to 72 mg/dl in 1999. For the TG, the range of geometric mean levels shrank from 253.5 mg/dl in 1998 to 154.1 mg/dl in 1999. For the HDLC, the range declined from 36 mg/dl at baseline to 31 mg/dl after one-year.

**Figure 4.**
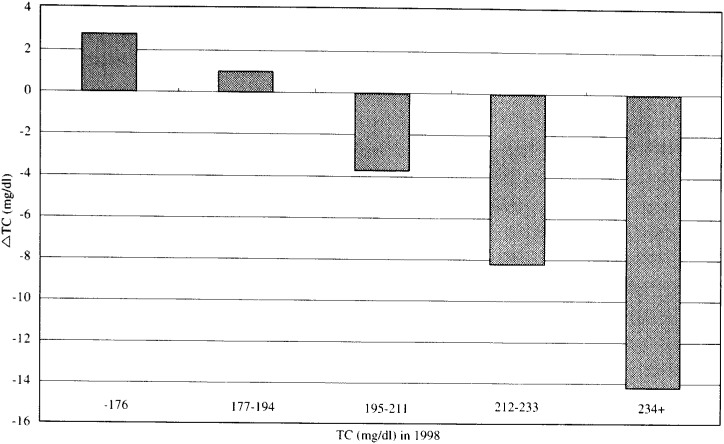
Adjusted mean levels of one - year change in TC by quintiles of the level in 1998. TC : Total cholesterol level.

**Figure 5.**
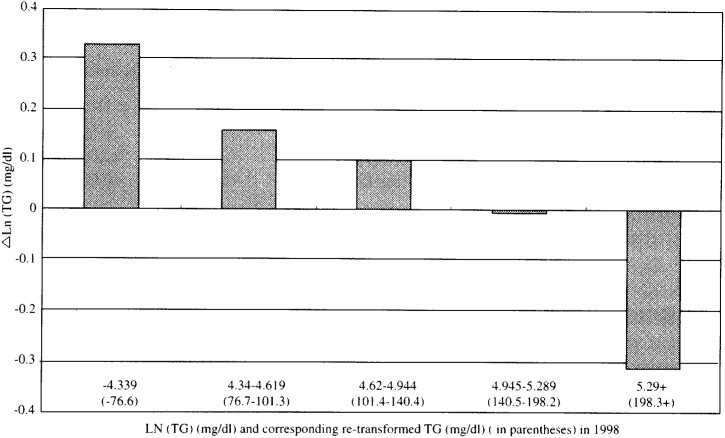
Adjusted mean levels of one - year change in Ln(TG) by quintiles of the level in 1998. Ln(TG) : Natural logarithm of triglycerides level.

**Figure 6.**
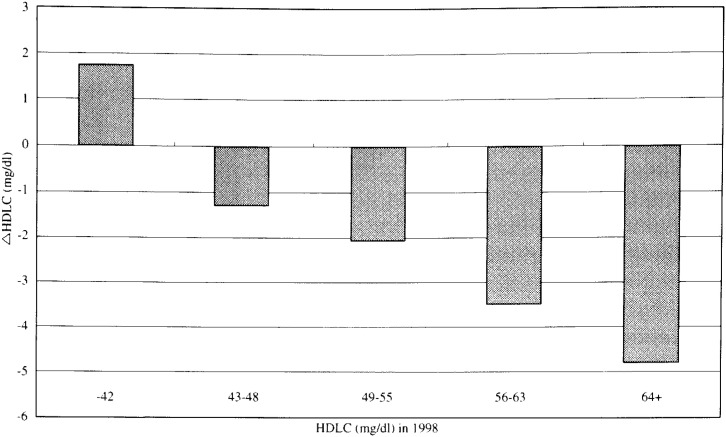
Adjusted mean levels of one - year change in HDLC by quintiles of the level in 1998. HDLC : High - density - lipoprotein cholesterol level.

In Fig. 4, 5, and 6, the adjusted mean levels of yearly change (Δ value) in each serum lipid are shown by quintiles of baseline level (the level in 1998) of the serum lipid. Clear inverse associations between Δ value and the baseline level were noted for any of the three lipids. These findings reveal that the confounding by age, BMI, ΔBMI, drinking or smoking history (i.e. the variables unadjusted for in Fig. 1, 2, and 3) cannot account for the shrinkage of range of measured values shown in Fig. 1, 2 and 3. Again, for not only the TC but also the TG or the HDLC, the ‘regression to the mean’ effects were shown, as characterized by the findings that the mean ΔTC, ΔLn(TG) and ΔHDLC levels steadily decreased with increasing lipid levels in 1998 (p for trend < 0.001 for any of ΔTC, ΔLn(TG) and ΔHDLC).

In Table 2 and 3, the results from the simple regression analyses and the multiple linear regression analyses using the baseline lipid levels as continuous variables (i.e. the latter model described in “Methods”) are summarized. For the ΔTC level as a dependent variable, the simple and partial regression coefficients (b and β_1_, respectively) for the TC level in 1998 were -0.22 (p<0.001) and -0.21 (p<0.001), respectively. Similarly, for ΔLn(TG) and ΔHDLC levels also, b and β_1_ values for the baseline lipid level were significantly negative (b= -0.35 (p<0.001) and β_1_ = -0.39 (p<0.001) for Ln(TG) level in 1998, and b= -0.13 (p<0.001) and β_1_ = -0.15 (p<0.001) for HDLC level in 1998, respectively).

**Table 2.** Simple regression coefficient (b) in each simple regression model including the baseline level of a serum lipid as an independent variable for change in the serum lipid level during one year (ΔTC, ΔLn(TG), or ΔHDLC) as a dependent variable.

Dependent variables	Independent variables	b	p value
ΔTC(mg/dl)	TC(mg/dl) in ’98	-0.22	<0.001
ΔLn(TG) (mg/dl)	Ln(TG) (mg/dl) in ’98	-0.35	<0.001
ΔHDLC (mg/dl)	HDLC (mg/dl) in ’98	-0.13	<0.001

TC: Total cholesterol level, Ln(TG): Natural logarithm of triglycerides level,

HDLC: High-density-cholesterol level

**Table 3.** Partial regression coefficients (β) in the multiple linear regression models including six independent variables for changes in serum lipid levels during one year (ΔTC, ΔLn(TG), ΔHDLC) as dependent variables.

Dependent variables	Independent variables	β		p value
ΔTC(mg/dl)	TC(mg/dl) in ’98	β_1_:	-0.21	<0.001
	Age in ’98*	β_2_:	-0.12	0.314
	BMI(Kg/m^2^)**	β_3_:	0.03	0.926
	ΔBMI(Kg/m^2^)**	β_4_:	2.5	0.02
	Drinking score***	β_5_:	0.25	0.768
	Smoking score***	β_6_:	0.08	0.937

ΔLn(TG) (mg/dl)	Ln(TG) (mg/dl) in ’98	β_1_:	-0.39	<0.001
	Age in ’98*	β_2_:	0.01	0.033
	BMI(Kg/m^2^)**	β_3_:	0.01	0.152
	ΔBMI(Kg/m^2^)**	β_4_:	0.04	0.061
	Drinking score***	β_5_:	0.02	0.235
	Smoking score***	β_6_:	0.06	0.007

ΔHDLC (mg/dl)	HDLC (mg/dl) in ’98	β_1_:	-0.15	<0.001
	Age in ’98*	β_2_:	0.08	0.032
	BMI(Kg/m^2^)**	β_3_:	-0.12	0.266
	ΔBMI(Kg/m^2^)**	β_4_:	-0.65	0.064
	Drinking score***	β_5_:	0.19	0.497
	Smoking score***	β_6_:	-0.56	0.113

Unit of β: * : mg/(dl × year), ** : mm^2^/dl, *** : mg/dl

TC : Total cholesterol level, Ln(TG) : Natural logarithm of triglycerides level,

HDLC : High-density-cholesterol level, BMI : Body mass index

## DISCUSSION

The intra-individual changes in bio-markers between annual periodic health check-ups are of great importance for the correct evaluation on individual trend in health status. However, the magnitude of the change can be determined not only by the real biological changes but also by the measurement error of the measured values and physiological fluctuation (seasonal or diurnal variation) of the value. The measurement error in itself corresponds to random error by which the measurement value is not deviated toward either side of the ‘true’ value (higher or lower area). Therefore, the measurement value reflects the ‘true’ value being located at either side of the measurement value. However, if the measurement value shows a symmetric distribution with one peak such as the Gaussian distribution, the expected frequency for the location of the ‘true’ value would not be equal between higher and lower area of the measurement value, under the condition that the measurement value is not equivalent to the mean value of the population. If the measurement value is located above the mean value of the population, the ‘true’ value is more likely to be located in lower area than in higher area. Conversely, the measured value being located below the mean value of the population more likely reflect higher true value rather than lower true value. Therefore, the measurement error more often leads to the over-estimation of the ‘true’ value rather than the underestimation when the measured value exceeds the mean of the population, while it more often brings about the underestimation of the ‘true’ value rather than the overestimation when the measured value is below the mean. Such measurement error can be limited by repeating baseline measurements several times and by controlling the condition under which measurements are taken.

On the other hand, from the standpoint of probability, it can also be pointed out that the measured value is more often located near the maximum level rather than the minimum level of within-person physiological fluctuation curve, if the measured value is found to be above the mean of the population. Conversely, when the measured value is lower than the mean of the population, the value is likely to be more often situated near the bottom of the physiological curve rather than the top. While in the former case the measured value tends to decrease by the remeasurement after a particular time interval, in the latter case it tends to increase by the replicate measurement (i.e. The return to the usual value can be found.).

Accordingly, the measurement error and the physiological fluctuation may be major causes for the regression to the mean within the intra-individual changes in bio-markers during a particular time interval. Moreover, the ‘regression to the mean’ effect resulted from these unintentional factors may inevitably introduce the regression dilution bias for risk assessment in a cohort study setting the bio-markers as risk factors at baseline.

As a result of the present study, such ‘regression to the mean’ effects could be found for any of the three serum lipids. The shrinkages of the range between the mean values of the top and bottom quintiles of the baseline level in each serum lipid were clearly noted after one-year interval. Clarke^1^^)^ suggested that the ratio of the range observed after a follow-up to the range at the baseline corresponds to the nonparametric estimate of the ‘regression dilution ratio’. According to this concept, the ‘regression dilution ratios’ after one-year interval were 0.78(=72/92) for the TC, 0.64(=l.073/1.665) for the Ln(TG), and 0.86(=31/36) for the HDLC, respectively.

However, of course, the intra-individual changes can also derive from the real biological changes. And there are many known exogenous factors contributing to the changes in serum lipid levels. Particular nutrients intake^13^^)^ obesity^14^^)^, exercise^15^^)^, alcohol intake^16^^)^ and smoking^17^^)^ are all important determinants of either or all of the TC, the TG and the HDLC. Of these factors, those being associated both with the baseline lipid level and subsequent changes in the lipid level can also bring about the ‘regression to the mean’ effect. Baseline level-dependent life style changes resulting in modification of changes in serum lipid levels can be included into these factors. For example, the individuals for which hypercholesterolemia was found at a health check-up, who tend to refrain from taking animal lipid-rich foods or make efforts for body weight reduction, are usually likely to lower the TC level during the following period.

In this study, the simple regression analysis was conducted to confirm the inverse association between the baseline level of serum lipid and subsequent yearly change in the level in each of the three lipids. Moreover, it was examined by a multivariate analysis whether the ‘regression to the mean’ effect still remained after excluding the effects of age, BMI at baseline, yearly BMI change, drinking score and smoking score. The results showed that for not only the TC but also the Ln(TG) and the HDLC, there were clear inverse associations between the baseline level of serum lipid and yearly change in the lipid level with and without considering the effects of these factors. According to a multiple linear regression analysis, partial regression coefficients for the baseline lipid level (β_1_) were -0.21 for the TC, -0.39 for the Ln(TG) and -0.15 for the HDLC, respectively. Interestingly, these values were quite similar to the simple regression coefficients (b) (-0.22 for the TC, -0.35 for the Ln(TG) and -0.13 for the HDLC, respectively). This similarity may reveal that the influences of the aforementioned possible confounding factors were relatively small. These β_1_ values were all significantly smaller than zero, suggesting that the ‘regression to the mean’ effect is an important component of causes for intra-individual changes in the serum lipid level during one year. The β_1_ value denotes the magnitude of the change in Δ value (yearly change in serum lipid level) corresponding to one unit (1mg/dl) change in the baseline level of the serum lipid after the exclusion of the effects of age, BMI, yearly BMI change, drinking score and smoking score. Therefore, it may fairly well represents the magnitude of the ‘regression to the mean’ effect except the real biological changes for the serum lipids.

If a measured value of the serum lipids showed a symmetric distribution with one peak such as the Gaussian distribution and the mean values did not change over the follow-up period, the Δ value of the individual having just the mean value would have been zero. However, in this study, the baseline levels corresponding to ‘Δ =0’ were supposed to be lower than the mean values in 1998 for the TC and the HDLC (Fig. 1, 3, 4, and 6). Two major reasons could be considered for these phenomena. One is that, as a whole, the measured value of the TC or the HDLC decreased during one-year with clear shrinkage of range. The other reason is that the distribution of the TC level or the HDLC level was not exactly identical with the Gaussian distribution but slightly skewed toward the lognormal distribution.

The reason why the mean TC level remarkably decreased between 1998 and 1999 could not be made clear, although occurrence of systematic measurement error and overall changes in dietary habits among the workers in this company could be considered as possible causes. However, with regard to the latter factor, at least, any intensive health education programs were not administered for the subjects during this period. At any rate, this decrement is unlikely to have basically biased the association between the baseline level and subsequent yearly change in the TC.

In this study, the follow-up period for the subjects was only one year. Clarke indicated from the Framingham and Whitehall study^1^^, ^^18^^)^ that the range of mean values in the baseline-defined groups for blood pressure and cholesterol became progressively smaller with longer intervals of follow-up. Accordingly, a longitudinal study with longer follow-up period would reveal quite different results (probably with more extreme shrinkage of the range). Nevertheless, the short follow-up period of this study could exclude several factors possibly threatening the validity of the study. For example, the effect of aging was negligible in the present study. Moreover, the selection bias resulting from the exclusion of early death and individuals with early occurrence of severe diseases, namely the survival effect^19^^)^, could also be considered minimal in this study.

As one problem corresponding to the limitation of this study, it can be pointed out that the subjects of this study were confined to middle or old-aged Japanese males. It is unclear whether the results of this study can also be applied to other populations. However, because the phenomenon of the regression to the mean is in itself derived not from the biological background but from the statistical background, we believe that the ‘regression to the mean’ effect shown in the present study is similarly seen regardless of gender, race or age of the objective population. Indeed, one-year change in serum cholesterol level in Clarke’s study^1^^)^ showed the effects almost similar to our results. Nevertheless, we emphasize that further investigations with a larger population size and a longer follow-up period are necessary for more detailed assessment of the ‘regression to the mean’ effect.

In conclusion, the results of the present study suggest the necessity of allowing the ‘regression to the mean’ effect for the correct evaluations on yearly changes in serum lipid levels at annual health check-ups of worksite. Moreover, the findings of this study also reveal that the relative risk calculated from a cohort study setting serum lipid levels as risk factors at baseline should be adjusted for regression dilution bias in many cases.
